# MO-GCAN: multi-omics integration based on graph convolutional and attention networks

**DOI:** 10.1093/bioinformatics/btaf405

**Published:** 2025-07-22

**Authors:** Yifan Dou, Golrokh Mirzaei

**Affiliations:** Department of Computer Science and Engineering, Ohio State University, Columbus, OH 43210, United States; Department of Computer Science and Engineering, Ohio State University, Columbus, OH 43210, United States

## Abstract

**Motivation:**

Cancer subtypes play a critical role in disease progression, prognosis, and treatment, making their detection essential for tailoring precision medicine. Studies have shown that multi-omics integration outperforms single-omics approaches in cancer subtyping tasks. However, due to the high-dimensionality of multi-omics data, many existing studies either fail to capture the correlation between true labels and learned features, or lack sufficient capacity to model complex biological representations. These limitations hinder the full potential of leveraging the rich and complementary information embedded in multi-omics datasets.

**Result:**

We propose a framework that leverages supervised feature learning and classification based on a graph-based learning approach with attention mechanism for cancer subtyping. More specifically, we train graph convolutional network models on each omics dataset to extract latent representations, which are then concatenated to form a comprehensive multi-omics feature embedding. We further develop sample fusion network based on the omics-specific graphs, incorporating the derived features and feeding them into a graph attention model for subtype classification. This two-stage multi-omics framework is applied to eight cancer types, with performance evaluated in terms of test accuracy, training time, macro-averaged precision, recall, and *F*-score. Experimental results show that the proposed method outperforms state-of-the-art approaches across various cancer types. Additionally, we provide empirical evidence supporting the hypothesis that retaining a limited number of high-confidence edges and utilizing enriched embeddings from intermediate graph neural network layers can improve predictive performance.

**Availability and implementation:**

Data and the code are available at https://github.com/YD-00/MO-GCAN-Updated.git.

## 1 Introduction

Cancer is a heterogeneous and complex disease, comprising many types and subtypes characterized by distinct biological features (Fisher *et al.* 2013, Baul *et al.* 2022). Various cancer subtypes may exhibit different responses to the same treatments (Blows *et al.* 2010, Krzyszczyk *et al.* 2018, Baul *et al.* 2022), emphasizing the importance of early subtype identification for designing appropriate and effective treatment strategies. Advancements in high-throughput sequencing technology have led to the emergence of several open-source omics datasets (The Cancer Genome Atlas Research Network 2011, The Cancer Genome Atlas Network 2012, The Cancer Genome Atlas Research Network et al. 2013), enabling development of models to automate cancer subtyping. Given that multiple omics platforms offer complementary insights from various molecular perspectives (Huang *et al.* 2017, Subramanian *et al.* 2020), successful integration is anticipated to yield a superior or at least equivalent prediction outcomes compared to single-omics data (Raj and Mirzaei 2022). In this article, we employ this criterion to gauge the effectiveness of integration.

A major challenge in multi-omics integration is the curse of dimensionality (Berisha *et al.* 2021), which arises due to the exponential increase in data dimensions as additional omics layer incorporated. This can lead to computational inefficiencies, increased noise level, and challenges in interpreting results. Additionally, the escalated model complexity can lead to overfitting (Hawkins 2004), where the model performs excessively well on training data but fails to generalize to unseen data, undermining the reliability of prediction. To address this problem, Chaudhary *et al.* (2018), Xu *et al.* (2019), Li *et al.* (2022), and Khadirnaikar *et al.* (2023) employed an autoencoder or stacked autoencoders to compresses the original data into a lower-dimensional space while preserving the original data representation by minimizing the reconstruction loss. Ye *et al.* (2023) and Raj *et al.* (2024) utilized Principal Component Analsysis (PCA) for feature reduction. While unsupervised methods in feature learning are widely used in studies, they have a significant limitation. Since unsupervised feature learning cannot distinguish which features are noise or ineffective for the prediction task. To address this issue, techniques such and MOGONET (Wang *et al.* 2021) incorporate supervised feature learning in their models. MOSAE (Tan *et al.* 2020) enhances the autoencoder by combining the prediction loss and the reconstruction loss, introducing a parameter to balance the tradeoff between the two loss functions. On the other hand, MOGONET (Wang *et al.* 2021) employs omics-specific graph convolution network (GCN) models (Kipf and Welling 2017) to learn features and utilizes the label probability distribution as the learned features (referred as label space in this article) which will be used for final prediction. However, the label space has limited dimensionality, potentially hindering its ability to effectively capture and map representations from highly complex biological data.

Based on this observation, we propose a novel omics-specific GCN feature learning method that expands the size of representation utilizing the latent space rather than the label space. Following a similar approach proposed by Chen *et al.* (2020), we retrieve values from the middle layer, which increases the dimensionality of the learned features. This enables a more complex matrix representation of the processed genomics data, effectively addressing the issue of limited dimensionality. Additionally, we exploit the attention mechanisms using multi-head attention strategy (Vaswani *et al.* 2017, Veličković *et al.* 2018) to capture the importance and relevance of different nodes or edges in the network, in contrast to traditional graph-based methods (Mirzaei 2022). Instead of relying on a fixed affinity network throughout training, this approach utilizes attention mechanism to iteratively calculate and update the weight from the neighboring nodes. This enables the model to focus on specific parts of the graph that are more informative or influential for the given task. Furthermore, we developed sample similarity network for each omics. The network was incorporated into the GCN specific features, and both the features and similarity network fusion (SNF)-based fused similarity (Wang *et al.* 2014) were used as inputs for the graph attention network (GAT) model (Veličković *et al.* 2018) for final classification. This is partly motivated by prior work leveraging similarity networks in gene-level analysis (Mirzaei 2023). Several successful practices of GAT on multi-omics integration or subtype classification such as omicsGAT (Baul *et al.* 2022) and MOGAT (Tanvir *et al.* 2024) demonstrate great potential of GAT model on the subtype task. Therefore, we propose a framework that applies omics-specific GCN models to learn from features with label supervision and utilizes a GAT model to adaptively learn from multiple data sources and make a final prediction.

The novelty of this work is summarized as follows: (i) We introduce a two-stage graph-based approach that integrates supervised feature learning followed by classification task by exploiting graph attention, convolutional network, and similarity network fusion. (ii) The proposed approach utilizes truth labels to guide the dimensionality reduction process while preserving sufficient representational capacity to map the complex input data, enabling more effective feature learning and subtype prediction. (iii) The model incorporates a selection from four types of omics including transcriptomics, proteomics, epigenetics, and genomics for classification task and achieves competitive performance compared to state-of-art approaches on most of the evaluated datasets. (iv) We demonstrate the effectiveness of using intermediate graph representation as learned features and retaining the strongest connections to optimize the performance through empirical studies. This study represents a significant step forward in leveraging advanced computational techniques to improve cancer subtype prediction through multi-omics integration. By integrating diverse omics data and incorporating graph-based methods, our approach offers new insights into the complex biological mechanisms underlying cancer.

## 2 Materials and method

Our proposed model consists of two main modules: (i) supervised feature learning module developed based on GCN (Kipf and Welling 2017); (ii) attention-based classification module developed based on SNF (Wang *et al.* 2014) and GAT (Veličković *et al.* 2018). In the feature learning stage, we utilize GCN models due to their stability in learning broadly and uniformly from all neighbors, making them well-suited for generalized feature representation. For the final classification stage, we employ GAT models, which use multi-head attention mechanisms to emphasize key features and enhance performance. This complementary design allows each model to leverage its strengths effectively. Our experiments validate this approach, confirming that combining GCNs for feature learning and GATs for classification effectively leverages the strengths of both models.

**Figure 1.**
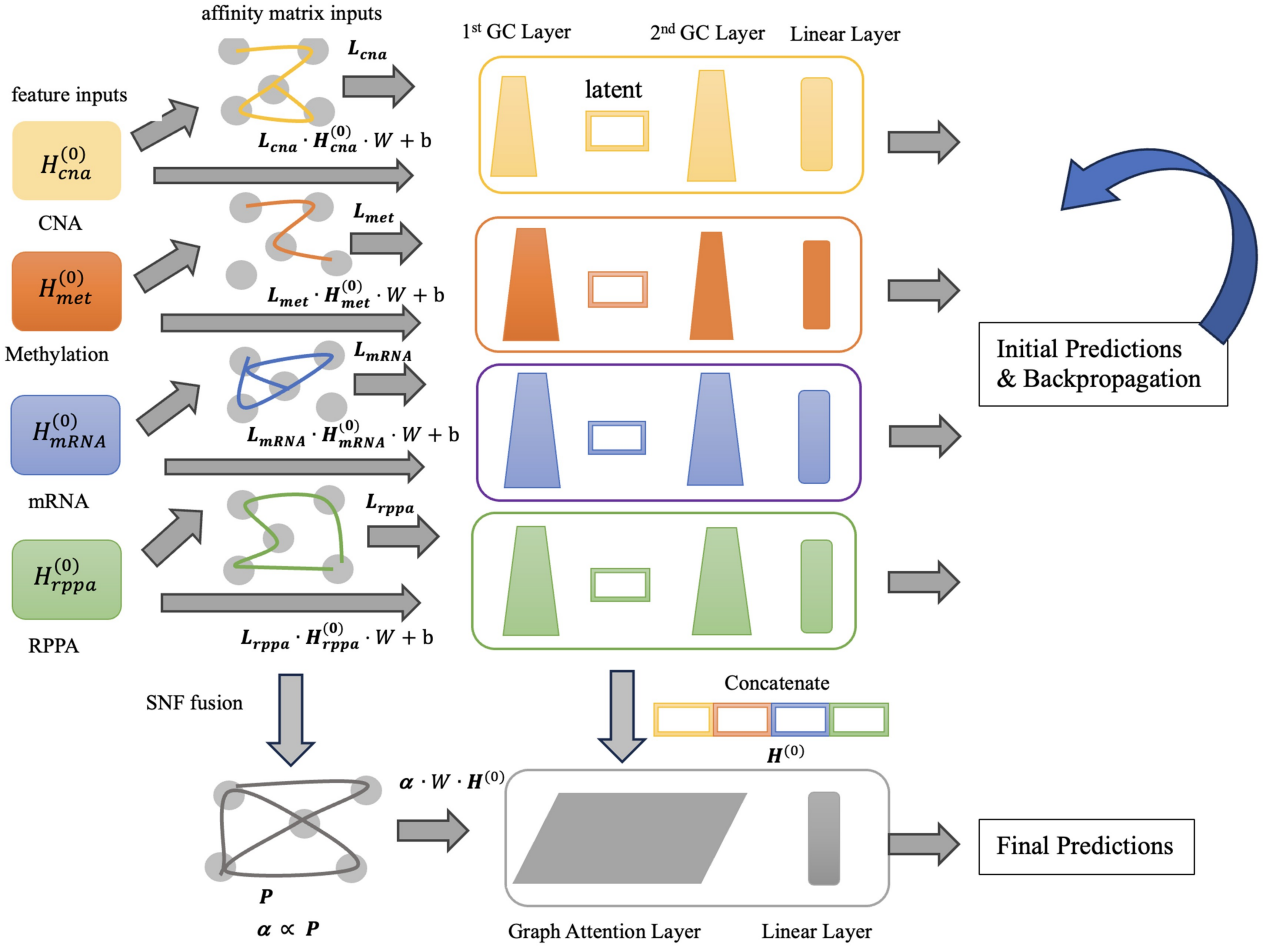
The overall framework of the proposed architecture (MO-GCAN). The model consists of (i) a feature learning module based on omics-specific GCNs. The GCN model is created using two types of inputs: the omics feature matrix and the graph structure affinity matrix. (ii) The classification module utilizes the similarity network matrices from the omics to create a fused SNF network and concatenates the features matrix from the first convolutional layer of the trained GCN model. A graph attention network is employed to prioritize the importance and relevance of features and make final prediction.

The overall architecture of the framework is shown as Fig. 1. In the feature learning module, we establish the graph structure by constructing an affinity matrix $L_{\mathrm{omics}\;}$ for each omics, and we utilize both the processed omics data $H_{\mathrm{omics}}^{\left(0\right)}$ as feature matrix and the affinity matrix $L_{\mathrm{omics}\;}$ to train GCN models. Once the omics-specific GCN models are trained, we forward the input omics data through the respective model until reaching the first convolutional layer, whose output serves as the latent data. This representation captures omics-specific features and reinforces the correlation between the learned features and label information, as it is an intermediate state between the input and the subtype.

The transition from the feature learning phase to the classification phase involves integrating input data from multiple omics into a fused network P using SNF (Wang *et al.* 2014). At the same time, we consolidate the feature matrix $H^{0}$ by concatenating the latent data produced by the selected omics-specific GCN models. In the classification module, both the SNF-fused similarity matrix P and the concatenated feature matrix $H^{0}$ are input into the GAT model for the final prediction. To optimize predictive accuracy, we propose a dynamic threshold selection algorithm to retain only the strongest connections. Subsequent experiments validate that carefully selecting edges contributes to achieving near-optimal results.

### 2.1 Supervised feature learning module based on omics-specific GCN

In feature learning module, we employed GCNs for omics-specific learning. Unlike traditional GCN models that primarily focus on semi-supervised learning—where labels from labeled data are propagated to unlabeled ones—our approach emphasizes on supervised learning. This is essential for clinical applications, as it enables the direct application of the trained GCN model to predict outcomes for new samples, even when their data were not available during the training process (Wang *et al.* 2021).

In the proposed graph structure, each node represents a patient, and edges quantify the strength of connections between patient pairs. Our GCN model consists of two stacked convolutional layers followed by a linear layer, trained using single-omics data. At the initial stage, the input omics data is utilized as node attributes, while the graph structure is defined by the affinity matrix. The value of the affinity matrix A between two patients $x_{i}$ and $x_{j}$ is calculated using a scaled exponential similarity kernel (Wang *et al.* 2014):

(1) $$A(i,j)=\;\exp(-\frac{\rho^{2}(x_{i},x_{j})}{\mu \nu_{i,j}})\;$$

where μ is a parameter that can be empirically set and $\nu_{i,j}$ is the scaling factor, defined as follows (Wang *et al.* 2014):

(2) $${\;v}_{i,j}=\frac{\mathrm{mean}(\rho (x_{i},N_{i}))+\mathrm{mean}(\rho (x_{j},N_{j}))+\rho (x_{i},x_{j})}{3}\;$$

where $N_{i}$, $N_{j}$ represent the neighbors for patients $x_{i},\;x_{j}$, respectively. We can define the number of neighbors as k. The scaling term $\nu_{i,j}$ is based on the mean of their k-nearest squared Euclidean distance ρ for patient $x_{i}$ and $x_{j}$ with their neighbors, together with the distance ρ between the two patients. The squared Euclidean distance ρ is defined as the sum of squared distances between a pair of patients $x_{i}$ and $x_{j}$ across all features m, and d represents the total number of features as:

(3) $$\rho (x_{i},x_{j})=\sum_{m=0}^{d}{\left(x_{i}^{m}-x_{j}^{m}\right)}^{2}\;$$

The resulting affinity matrix A is an n×n matrix, where each element represents the similarity between a pair of patients, thereby determining the strength of their connection. Since GCN models rely on message passing to iteratively aggregate information from connected nodes, identifying meaningful connections is essential. To ensure effective information sharing and updating, we set a proper threshold ε to filter out weak connections. Any value in A(i,j) below ε is set to 0, retaining only strong connections.

(4) A(i,j)={0 if A(i,j)<ε A(i,j) else 

Next, we compute the normalized graph Laplacian L from the affinity matrix A (Li *et al.* 2022):

(5) $$L=D^{-1}A\;$$

where $D^{-1}$ represents the inverse of the degree matrix D derived from the affinity matrix A. Subsequently, we calculate the forward pass in GCN as (Li *et al.* 2022, Kipf and Welling 2017):

(6) $$H^{n+1}\;={\sigma (L\cdot H}^{\left(n\right)}\cdot W^{\left(n\right)}+b_{n})$$

This equation elucidates the mechanism of message aggregation and the integration of omics data with the affinity matrix. The Laplacian matrix L, derived from the affinity matrix A, serves as the graph structure, indicating both the connectivity between nodes (non-zero values) and the strength of those connections (numerical magnitude). Here, $H^{n+1}$ represents the output of the ${\left(n+1\right)}^{}th$ hidden layer, and $H^{0}$ denotes the input single-omics data. The parameter $W^{\left(n\right)}$ denotes the weight matrix for linear transformation in the $n^{}th$ layer, $b_{n}$ represents a bias term, and σ is activation function, specifically the exponential linear unit. Affinity values, which can be either be 0 or numerical, guide the aggregation of messages through connected nodes, allowing information to propagate effectively based on strength of the connections. The Laplacian matrix normalizes the propagation, ensuring a uniform and balanced distribution of information across the graph. Finally, the latent data are transformed from the size of hidden layer to the number of labels, as: 
 

(7) $$Y=W_{2}H+b_{2}$$

where $W_{2}$ is the weight matrix in the output layer, and Y is the label space, representing the probability distribution of the labels. The label with the highest probability is selected as the predicted outcome. From Equations (4)–(6), we can observe the significance of setting a threshold, as it greatly influences the multiplier that guides the direction and magnitude of the message passing during the forward pass. To optimize performance, we applied dynamic threshold selection approach (as described in the subsequent section) to retain the strongest connections among the nodes.

To generate the latent data for each omics, we load the base model including all the trained parameters W and b for each layer, forward the input omics data and its graph Laplacian matrix to the trained omics-specific model, and obtain the output of the first GC layer $H^{1}$ as the latent data for the single-omics. This corresponds to as specific instance for Equation (6) for the first hidden layer, as:

(8) $${\;H}^{1}={\sigma (L\cdot H}^{\left(0\right)}\cdot W^{\left(0\right)}+b_{0})$$

After obtaining the latent data from all omics, we select omics according to initial predictions and concatenate these outputs from the selected omics-specific GCN models into a unified matrix to form an integrated feature representation. Let n represents the number of samples, and h the size of the latent data for each single-omics type. As a results, if four omics are all selected, the concatenated matrix has dimensions n×4h, where the factor of 4 corresponds to the four omics data types. This process condenses the high-dimensional data into the size of the hidden layer while maintaining the relationship between the labels and the learned features. The parameters $W^{\left(n\right)}\;$and $b_{n}$ in Equation (6), which are derived through backpropagation of label information during GCN training, encapsulate valuable information about the true label. This enforces a strong correlation between the labels and the latent features, making it particularly significant in supervised feature learning.

### 2.2 Similarity network fusion

To construct a graph for the GAT model, we require both integrated feature values and integrated edge values. Similarity network fusion (SNF) (Wang *et al.* 2014) is a well-established technique for integrating similarity networks across patients from multiple data sources. This network-based method performs a nonlinear combination of similarity networks, making it highly effective at capturing complex relationships between nodes while retaining complementary information.

The normalized patient similarity is derived from Equation (9) (Wang *et al.* 2014), based on the calculated affinity matrix A, which represents the similarity between patient i and patient j. In this equation, the self-similarity is set to 0.5 as suggested by authors of SNF (Wang *et al.* 2014). The similarity between two distinct patients is determined by the ratio of their affinity value to the sum of affinity values from other pairs excluding the self-similarity, as:

(9) P(i,j)={A(i,j)2∑k≠iA(i,k), j≠i  12, j=i  

The local affinity (Wang *et al.* 2014) is calculated using the concept of *K*-nearest neighbors, as introduced in Equation (10). Unlike the patient similarity metric, which considers all pairs of patients, the local affinity only accounts for pairs within the *K*-nearest neighborhood. If two patients are not K-nearest neighbors, the local affinity between them is set to 0, as:

(10) S(i,j)={A(i,j)∑k∈NiA(i,k), j∈Ni  0 otherwise 

Using the patient similarity and local affinity networks, we can fuze the similarity networks from various omics source into a single network by iteratively updating the patient similarity matrix. Let $S^{u}$ represents an n×n local affinity matrix for n patients in the ${u\mathrm{th}}^{}$ omics, and $P^{k}$ represents an n×n patient similarity matrix for n patients from the other omics (k≠u). The patient similarity matrix for the ${uth}^{}$ omic is updated as follows:

(11) $$P^{\left(u\right)}=S^{u}\times (\frac{\sum_{k\neq u}P^{\left(k\right)}}{m-1})\times {{(S}^{u})}^{T},u=1,2,\ldots ,m\;$$

where m represents the total number of omics.

The fused network is then computed as the average of all the patient similarity matrices obtained from Equation (11), as:

(12) $$P=\frac{\sum_{u=1}^{m}P^{u}}{m}$$

This fused network P will be utilized to construct graph edges and combined with the concatenated feature matrix (from the omics-specific GCN models) as node features for the subsequent GAT-based classifier.

### 2.3 Attention based classification module using GAT

The GAT (Veličković *et al.* 2018) extends the GCN by introducing masked self-attentional layers. Instead of utilizing a fixed affinity matrix, GAT employs parameters to compute and iteratively update the affinity matrix, making the connections among nodes dynamic. The omicsGAT (Baul *et al.* 2022) framework applies GAT to cancer subtyping using RNA-seq data. Building upon this, our GAT model consists of a graph attention layer and a linear layer, integrating multi-omics data. Specifically, the concatenated latent data serves as the feature matrix, while the fused network P from Equation (12) is used as the graph structure data. The forward pass for a graph attention layer is formulated as (Veličković *et al.* 2018, Tanvir *et al.* 2024):

(13) $${{\;h}_{i}}^{n+1}=\sigma (\sum_{j\in N_{i}}\alpha_{ij}W{h_{j}}^{n})$$

where $h_{i}^{n+1}$ is the value of node i at the $({n+1)\mathrm{th}}^{}$ layer, $h_{j}^{n}$ denotes the value of a neighboring node j at the ${\mathrm{nth}}^{}$ layer, W is a learnable weight matrix, and ${\;N}_{i}$ is the set of neighbors for the node i. The fused network P defines whether the node j is considered a neighbor of the node i. Information sharing and impact between nodes i and j occur only when P(i,j) has a non-zero value. A major difference between GAT and GCN lies in the forward pass. While GCN directly uses the edge matrix as a multiplier as in Equation (6), GAT uses the attention coefficient $\alpha_{ij}$, based on the fused network P and other weight parameters, as a multiplier. This enables neighboring nodes to exert varying and dynamic influences on the center node, thereby enhancing learning precision. To stabilize performance, we apply multi-head attention by concatenating the output of two single-head attentions.

The attention coefficient $\alpha_{ij}$ (Veličković *et al.* 2018, Tanvir *et al.* 2024) between nodes i and j is calculated as:

(14) αij={exp⁡(LeakyReLU(aT* [Whi∥ Whj]))∑r∈Niexp⁡(LeakyReLU(aT*[Whi∥ Whr])), P(i,j)>0 0    otherwise 

where symbols ∥ and $\cdot^{T}$ represent concatenation and transposition operations, respectively, and a is a learnable weight vector. We then use a SoftMax function to determine the importance of node i to node j (Baul *et al.* 2022). The linear layer processes the output of the graph attention layer to generate a label probability distribution. The label with the highest probability is selected as the predicted outcome, as:

(15) $$H_{\mathrm{cls}}=\mathrm{Softmax}(W_{\mathrm{cls}}H_{in}+b_{\mathrm{cls}})\;$$

where $H_{\mathrm{cls}}$ is the output of the classifier, $W_{\mathrm{cls}}$ is the learnable weight in the output layer, $H_{in}$ is the input to the final layer (i.e. the output of the graph attention layer), and $b_{\mathrm{cls}}$ is a bias term in the output layer.

## 3 Experiments and results

### 3.1 Data processing

We downloaded and processed the multi-omics data including copy number alteration (CNA) (Mirzaei and Petreaca 2022), DNA methylation, mRNA-sequencing, and reverse phase protein array (RPPA) from eight cancer types from the cbioportal website: https://www.cbioportal.org/ (Cerami *et al.* 2012), as summarized in Table 1. For cases with duplicate sample IDs, we retained the most recent data entry and included only samples with available subtype labels. Samples lacking any omics data were excluded from the analysis. To address missing values, we removed features with more than 10% missing data (*NA* values) and replaced the remaining *NA* values with 0. Table 1 provides an overview of the data distribution across the eight cancer types after data preprocessing. Each row represents a specific cancer type, with the first four columns indicating the number of features for each omics type. The last two columns display the total number of classes and their distribution, respectively.

**Table 1.** The size of processed omics data and the label distribution.^a^

Cancer datasets	CNA	Methylation	mRNA	RPPA	# of classes	Class distribution
TCGA-LGG	19 453	22 589	20 164	191	3	(204, 145, 72)
TCGA-UCEC	17 913	22 599	17 508	192	4	(128, 119, 116, 42)
TCGA-STAD	24 481	22 583	16 766	194	5	(178, 62, 39, 26, 6)
TCGA-SARC	24 752	22 539	20 155	194	4	(75, 63, 42, 14)
TCGA-COADREAD	23 331	22 585	17 508	192	8	(175, 77, 49, 45, 7, 5, 3, 2)
TCGA-CESC	24 158	22 565	20 021	194	2	(127, 21)
TCGA-HNSC	25 129	22 587	20 224	192	2	(186, 16)
TCGA-BRCA	23 706	22 593	20 212	191	5	(381, 167, 141, 71, 27)

a Number of features are shown in CNA, methylation, mRNA, and RPPA columns.

### 3.2 A case study: brain lower grade glioma cancer data (TCGA-LGG)

We used the experiment with brain lower grade glioma cancer dataset (TCGA-LGG) as a case study to illustrate how MO-GCAN and the dynamic threshold selection approach work. The framework was developed and tested using Python libraries, including Torch, NumPy, and Pandas. To ensure reproducibility, we fixed the random seed for both Torch and NumPy. The dataset was preprocessed and split into training and testing sets in a 75%–25% ratio. To compute the affinity matrix for each omics dataset, we set the number of neighbors K to 20, and the scaling parameter μ to 0.5. Each omics-specific dataset was then used to train a GCN model, generating latent data and initial predictions. The GCN model parameters includes number of neurons for each hidden layer as 100, dropout rate as 0.5, Adam optimizer with a learning rate of 0.001 and a weight decay rate of 0.01, and cross-entropy loss function.

**Figure 2.**
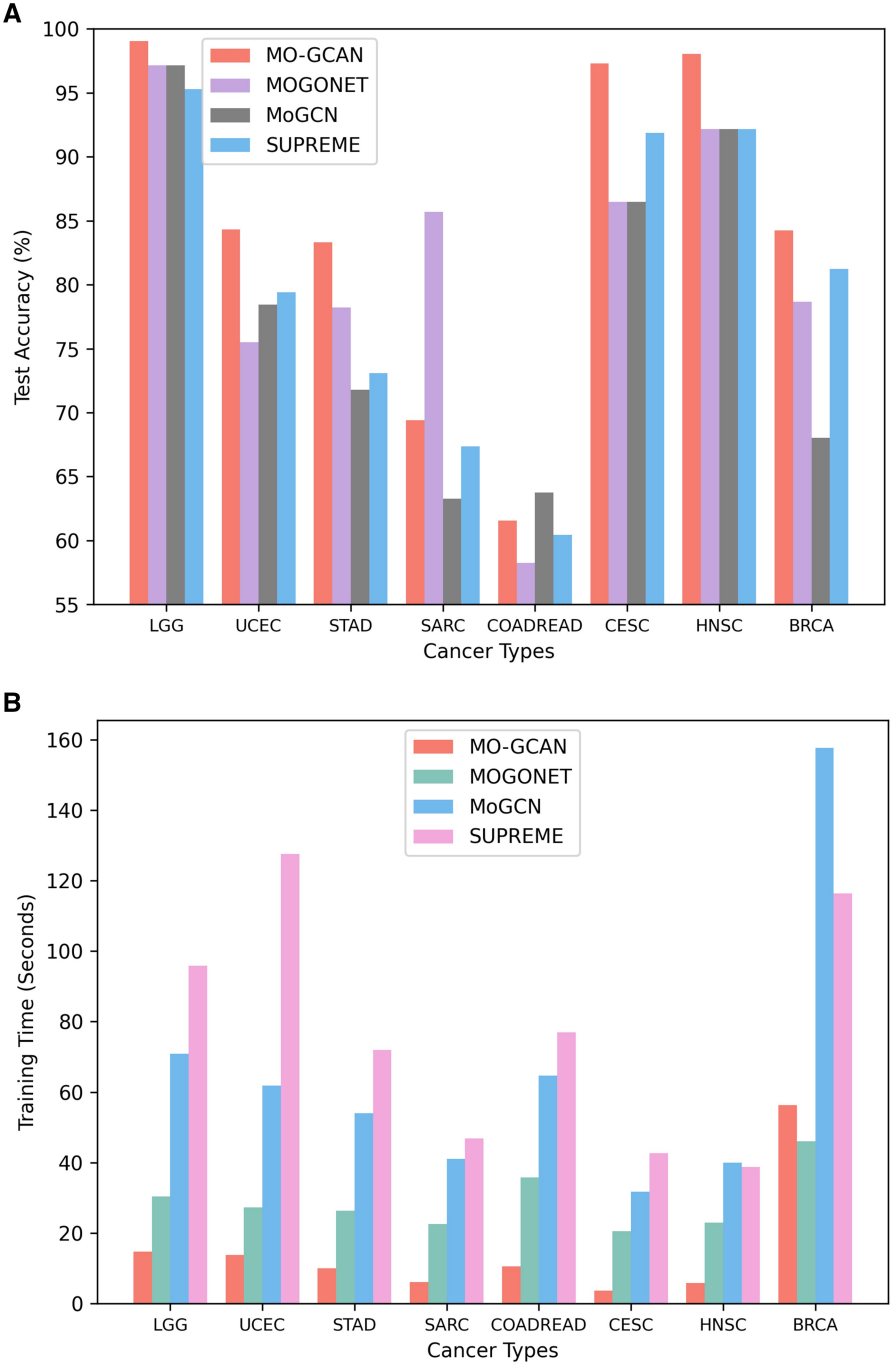
Comparison of test accuracy and training time between proposed approach (MO-GCAN) and state-of-art methods (MOGONET, MoGCN, and SUPREME).

Motivated by the work of Cao *et al.* (2024) on determining the optimal number of nodes for effectively representing graph networks, this study investigates how the performance varies with the number of edges in the context of high-dimensional multi-omics data. As Figs. 1 and 2, available as supplementary data at *Bioinformatics* online, show, the performance of GNN could be unstable depending on the choice of thresholds in Equation (4). Specifically, there is an overall trend of performance declining as more connections are retained—starting from a near-minimum threshold of at least 1%, increasing in steps of 10%. To optimize the model performance, we proposed an algorithm for dynamic threshold selection. The main idea about this algorithm is to find a minimum percentage of remained edges without arising a ‘singular matrix’ error. This error arises when det⁡(D)=0, making the degree matirx D in Equation (5) non-invertible. A threshold is calculated by a percentage to select the top strongest connections, arranged in descending order of connection values. For example, setting a percentage at 0.01 retains the top 1% of values in the affinity matrix, which equates to 421 ×421×1% ≈ 1772 connections for the LGG dataset.

After detecting the near-minimum threshold and a trained omics-specific model for each omics dataset, we forwarded the processed omics data and an affinity network to the chosen omics-specific GCN model to generate latent data for the selected omics. We then concatenated the selected latent data, constructed a fused similarity, detect a near-minimum threshold for the fused network to filter out weak connections, and put them to a graph attention network that employs two-head attention mechanism with the cross-entropy loss function. The model attained an accuracy of 99.06%, surpassing all initial single-omics predictions and demonstrating the effective integration of multi-omics data.

We summarized our proposed algorithm for dynamic threshold selection as below. Since we did not know the data distribution beforehand, we used percentages to calculate the concrete threshold values for Equation (4) to filter out weak edges. The dynamic threshold selection algorithm was blinded with our proposed Mo-GCAN approach in both stages, and the results demonstrating our framework with this optimization algorithm perform competitively across the majority of the evaluated datasets.

Proposed algorithm for dynamic threshold selection: To sparcify the adjacency matrix $A\in R^{n\times n}$ while ensuring numerical stability:

1. *Initialize the minimum percentage* p  *= 0.01*

*2. Sort all elements of* A  *in descending order*

*3. Compute threshold*  $\tau_{p}\;\mathrm{such}\;\mathrm{that}\;\mathrm{only}\;\mathrm{the}\;\mathrm{top}\;\left\lceil p\;.\;|\varepsilon |\right\rceil \;(\varepsilon \;\mathrm{denotes}\;\mathrm{the}\;\mathrm{number}\;of\;\mathrm{elements}\;in\;A)$

*4. Binarize* $A\;by\;\mathrm{setting}\;A_{ij}=1\;if\;A_{ij}\geq \tau_{p},\;\mathrm{and}\;0\;\mathrm{otherwise}$

*5. Construct the diagonal degree matrix* $D,\;\mathrm{where}\;D_{ij}=\sum_{j}A_{ij}$

*6. Check rank: if* D is not full rank, increment p←p+0.005* and repeat steps 3–6 with the original* ***A***

*7. The final percentage* $p^{*}\;$*defines the minimal threshold ensuring* D  *is full-rank, avoiding downstream singularity issue*

### 3.3 Evaluating Mo-GCAN across various cancer types

To assess the effectiveness, robustness, and generalizability of our approach, we further tested MO-GCAN on eight different types of cancer datasets. The evaluation comprised the followings:

1. Comparison of latent space and label space: We aim to support our hypothesis that the label space, due to its limited dimensionality, may struggle to effectively capture and represent the complexity of high-dimensional biological data. In contrast, richer embeddings extracted from earlier hidden layers may better preserve this complexity and improve downstream performance. To test this, we conducted an experiment comparing prediction accuracy using embeddings retrieved from the first GCN layer, second GCN layer, and the final fully connected (FC) layer (i.e. the label space) within our MO-GCAN framework. As shown in Table 6, available as supplementary data at *Bioinformatics* online, the more enriched embeddings from the first and second layers yielded superior predictive performance, highlighting their potential in enhancing model accuracy.
2. Comparison of thresholds for retaining edges: We investigated our hypothesis that preserving only the strongest connections with at least 1% top edges can improve the performance of graph neural networks. Using our proposed dynamic threshold selection method, we determined the near-minimum percentage of edges to retain. Building on this, we experimented with five different retention levels, increasing the percentage to calculate the threshold in steps of 0.1. As shown in Figs. 1 and 2, available as supplementary data at *Bioinformatics* online, there is an overall descending trend of prediction performance as more edges involved, supporting the effectiveness of our approach.
3. Comparison with state-of-art approaches: We compared our proposed MO-GCAN approach with three state-of-the-art methods that also utilize graph neural networks as their backbone architectures, such as MOGONET (Wang *et al.* 2021), MoGCN (Li *et al.* 2022), and SUPREME (Kesimoglu and Bozdag 2023). As shown in Fig. 2, Figs. 4 and 5, available as supplementary data at *Bioinformatics* online, MO-GCAN is more time-efficient and achieves superior predictive performance across most datasets, with the exception of TCGA-SARC and TCGA-COADREAD. To account for class imbalance (Yang and Mirzaei 2024), we evaluated performance using macro-averaged precision, recall, and *F*1-score, as summarized in Table 2. For the majority of datasets, MO-GCAN outperformed the competing methods, with the best results highlighted in bold.
4. Comparison of GCN and GAT as the feature learning and classification modules: We conducted a comparison between different configurations of graph convolutional networks (GCNs) and graph attention networks (GATs) used as feature learning and classification modules, respectively. Specifically, we compared our proposed MO-GCAN approach with two alternative setups: “All GATs” (applying GAT as the feature learning module) and “All GCNs” (applying GCN as the classification module), as detailed in Tables 1–3, available as supplementary data at *Bioinformatics* online. Our observations indicate that GCNs provide more stable performance in feature extraction and initial predictions, while GATs show a slight advantage in enhancing final prediction outcomes.
5. Comparison with single-omics and multi-omics: One limitation of our work is the suboptimal integration of multi-omics data for several datasets. While we anticipated that multi-omics prediction would outperform—or at least match—the performance of single-omics prediction, results from Table 3, available as supplementary data at *Bioinformatics* online, show that, for five out of eight datasets, single-omics models achieved better results. Although our proposed MO-GCAN method outperforms some state-of-the-art approaches overall, this comparison highlights that more effective strategies for integrating multi-omics data are still needed to fully realize their potential.
6. Evaluation of the cause of the suboptimal performance: We presented the confusion matrices generated by MO-GCAN for each cancer type in Fig. 3, available as supplementary data at *Bioinformatics* online. These results reveal an additional perspective for potential improvement: for certain cancer types, such as TCGA-SARC and TCGA-COADREAD, our approach struggles to distinguish subtle differences between the two groups, and misclassifications are primarily concentrated between specific subtype pairs. This limitation likely contributes to the suboptimal performance of our method on these cancer types compared to state-of-the-art approaches, as shown in Table 2. Further investigation is needed to improve MO‑GCAN’s robustness and generalizability, especially for datasets where it struggles to distinguish certain pairs of subtypes.

**Table 2.** Comparison of metrics over multiple classes considering data imbalance between the proposed MO-GCAN approach and the baseline approaches (MoGCN, SUPREME, and MOGONET).^b^

Metrics for multiple classes	Cancer dataset	MO-GCAN (%)	MOGONET (%)	MoGCN (%)	SUPREME (%)
Macro avg precision	TCGA-LGG^a^	**99.12**	97.79	96.60	93.67
	TCGA-UCEC	81.45	66.37	**83.50**	79.83
	TCGA-STAD^a^	**62.48**	55.21	53.63	59.09
	TCGA-SARC	66.07	**78.87**	37.84	61.47
	TCGA-COADREAD	24.40	26.89	**38.21**	28.51
	TCGA-CESC^a^	**91.67**	43.24	43.24	81.72
	TCGA-HNSC^a^	**90.00**	46.08	46.08	46.08
	TCGA-BRCA^a^	**81.45**	73.96	56.32	65.27
Macro avg recall	TCGA-LGG^a^	**99.35**	97.54	96.84	95.29
	TCGA-UCEC^a^	**82.20**	66.06	71.60	72.33
	TCGA-STAD^a^	**65.50**	57.28	58.11	51.97
	TCGA-SARC	64.99	**74.10**	43.75	50.43
	TCGA-COADREAD^a^	**32.01**	25.37	27.45	31.21
	TCGA-CESC^a^	**98.44**	50.00	50.00	86.88
	TCGA-HNSC^a^	**98.94**	50.00	50.00	50.00
	TCGA-BRCA^a^	**81.68**	69.58	42.35	65.11
Macro avg *F*1-score	TCGA-LGG^a^	**99.23**	97.66	96.71	94.38
	TCGA-UCEC^a^	**81.73**	65.88	73.65	73.74
	TCGA-STAD^a^	**63.74**	54.74	55.61	53.95
	TCGA-SARC	62.56	**75.71**	38.39	50.86
	TCGA-COADREAD	27.23	24.90	26.71	**29.47**
	TCGA-CESC^a^	**94.66**	46.38	46.38	83.98
	TCGA-HNSC^a^	**93.91**	47.96	47.96	47.96
	TCGA-BRCA^a^	**80.40**	71.42	39.32	65.07

a Cancer types where our approach outperformed the comparison approaches.

b Results are based on selection from four omics: copy number alteration (genomics), DNA methylation (epigenomics), mRNA-sequencing (transcriptomics), and reverse phase protein array (proteomics).

Bold values indicate the best performance among the compared approaches.

## 4 Conclusion

In this article, we proposed a framework for supervised feature learning and classification utilizing graph convolutional, attention, and sample fusion networks. Specifically, we developed omics-specific GCNs and extract data from the GCN hidden layer to obtain dimension-reduced data, concatenate them from multi-omics data sources, and along with sample fusion network feed it to a GAT classifier to make a final prediction. We also adopted dynamic threshold algorithm and a more enriched embeddings retrieved from the middle layer to optimize the performance of GNN networks. The experimental results across eight TCGA cancer types demonstrated that for most datasets, the proposed approach is efficient, robust, and generalizable, and it is capable of achieving superior prediction performance with reduced training time compared to state-of-the-art approaches. This framework can be extended to various datasets and applied to different disease types, offering a promising tool for integrating multi-omics data in precision medicine.

## Supplementary Material

btaf405_Supplementary_Data

## Data Availability

The data and code underlying this article are available at https://github.com/YD-00/MO-GCAN-Updated.git
