# Supplementary material for: MO-GCAN: multi-omics integration based on graph convolutional and attention networks
Source: Bioinformatics. 2025 Jul 22;41(8):btaf405. doi: 10.1093/bioinformatics/btaf405 (PMC12342174; doi:10.1093/bioinformatics/btaf405)
Supplement: btaf405_Supplementary_Data [file btaf405_supplementary_data.pdf]

Supplementary Table 1. All GCNs. Comparison of accuracy (%) in initial omics-specific GCN predictions and final GCN predictions using all omics and selected omics. Results are based on a selection of four omics types: copy number alteration (genomics), DNA methylation (epigenomics), mRNA sequencing (transcriptomics), and reverse phase protein array (proteomics).

Cancer dataset	GCN_{CNA}	GCN_{met}	GCN_{mRNA}	GCN_{RPPA}	GCN_{all_omics}	Selected Omics	$GCN_{selected\_omics}$	Training Time (seconds)
TCGA-LGG	94.34	97.17	97.17	79.25	**98.11**	CNA, met, mRNA, RPPA	**98.11**	14.9
TCGA-UCEC	62.75	72.55	**89.22**	68.63	81.37	mRNA, RPPA	79.41	12.9
TCGA-STAD	65.38	76.92	76.92	65.38	**82.05**	CNA, met, mRNA, RPPA	**82.05**	9.9
TCGA-SARC	67.35	79.59	**83.67**	61.22	81.63	CNA, met, mRNA, RPPA	81.63	6.1
TCGA-COADREAD	54.95	**68.13**	61.54	49.45	67.03	CNA, met, mRNA, RPPA	67.03	10.6
TCGA-CESC	89.19	**97.30**	94.59	78.38	**97.30**	CNA, met, mRNA, RPPA	**97.30**	3.7
TCGA-HNSC	96.08	**100.00**	94.12	88.24	**100.00**	CNA, met, mRNA, RPPA	**100.00**	5.1
TCGA-BRCA	64.97	56.85	**87.82**	74.11	83.25	CNA, mRNA, RPPA	82.74	53.3

Supplementary Table 2. All GATs. Comparison of accuracy (%) in initial omics-specific GAT predictions and final GAT predictions using all omics and selected omics. Results are based on a selection of four omics types: copy number alteration (genomics), DNA methylation (epigenomics), mRNA sequencing (transcriptomics), and reverse phase protein array (proteomics).

Cancer dataset	GAT_{CNA}	GAT_{met}	GAT_{mRNA}	GAT_{RPPA}	GAT_{all_omics}	Selected Omics	$GAT_{selected\_omics}$	Training Time (seconds)
TCGA-LGG	92.45	**97.17**	96.23	74.53	**97.17**	CNA, met, mRNA, RPPA	**97.17**	**25.6**
TCGA-UCEC	50.00	29.41	**83.33**	63.73	63.73	mRNA, RPPA	60.78	23.0
TCGA-STAD	66.67	**80.77**	70.51	62.82	78.21	CNA, met, mRNA	73.08	19.1
TCGA-SARC	63.27	77.55	**85.71**	55.10	81.63	CNA, met, mRNA	61.22	12.1
TCGA-COADREAD	53.85	16.48	57.14	48.35	**67.03**	CNA, mRNA	62.64	21.5
TCGA-CESC	81.08	**94.59**	89.19	86.49	86.49	CNA, met, mRNA, RPPA	86.49	8.7
TCGA-HNSC	92.16	92.16	**94.12**	92.16	**94.12**	CNA, met, mRNA, RPPA	**94.12**	8.1
TCGA-BRCA	63.96	57.87	**87.31**	71.57	67.51	CAN, mRNA	61.42	78.4

Supplementary Table 3. MO-GCAN. Comparison of accuracy (%) in initial omics-specific GCN predictions and final GAT predictions using all omics and selected omics. Results are based on a selection of four omics types: copy number alteration (genomics), DNA methylation (epigenomics), mRNA sequencing (transcriptomics), and reverse phase protein array (proteomics).

Cancer dataset	GCN_{CNA}	GCN_{met}	GCN_{mRNA}	GCN_{RPPA}	GAT_{all_omics}	Selected Omics	$GAT_{selected\_omics}$	Training Time (seconds)
TCGA-LGG	94.34	97.17	97.17	79.25	**99.06**	CNA, met, mRNA, RPPA	**99.06**	14.7
TCGA-UCEC	62.75	72.55	**89.22**	68.63	81.37	mRNA, RPPA	84.31	13.8
TCGA-STAD	65.38	76.92	76.92	65.38	**83.33**	CNA, met, mRNA, RPPA	**83.33**	10.0
TCGA-SARC	67.35	79.59	**83.67**	61.22	69.39	CNA, met, mRNA, RPPA	69.39	6.1
TCGA-COADREAD	54.95	**68.13**	61.54	49.45	61.54	CNA, met, mRNA, RPPA	61.54	10.6
TCGA-CESC	89.19	**97.30**	94.59	78.38	**97.30**	CNA, met, mRNA, RPPA	**97.30**	3.7
TCGA-HNSC	96.08	**100.00**	94.12	88.24	98.04	CNA, met, mRNA, RPPA	98.04	5.8
TCGA-BRCA	64.97	56.85	**87.82**	74.11	83.25	CNA, mRNA, RPPA	84.26	56.3

Supplementary Table 4. Comparison in terms of accuracy (%) among the proposed MO-GCAN approach with selected omics and state-of-art approaches (MOGONET, MoGCN, and SUPREME).

Cancer dataset	Cancer type	MO-GCAN	MOGONET	MoGCN	SUPREME
TCGA-LGG	Brain Lower Grade Glioma	**99.06**	97.17	97.17	95.28
TCGA-UCEC	Uterine Corpus Endometrial Carcinoma	**84.31**	75.49	78.43	79.41
TCGA-STAD	Stomach Adenocarcinoma	**83.33**	78.21	71.79	73.08
TCGA-SARC	Sarcoma	69.39	**85.71**	63.27	67.35
TCGA-COADREAD	Colorectal Adenocarcinoma	61.54	58.24	**63.74**	60.44
TCGA-CESC	Cervical Squamous Cell Carcinoma and Endocervical Adenocarcinoma	**97.30**	86.49	86.49	91.89
TCGA-HNSC	Head and Neck Squamous Cell Carcinoma	**98.04**	92.16	92.16	92.16
TCGA-BRCA	Breast Invasive Carcinoma	**84.26**	78.68	68.02	81.22

Supplementary Table 5. Comparison in terms of training time (seconds) among the proposed MO-GCAN approach and state-of-art approaches (MOGONET, MoGCN, and SUPREME).

Cancer dataset	Cancer type	MO-GCAN	MOGONET	MoGCN	SUPREME
TCGA-LGG	Brain Lower Grade Glioma	14.7	30.4	70.9	95.8
TCGA-UCEC	Uterine Corpus Endometrial Carcinoma	13.8	27.3	61.9	127.6
TCGA-STAD	Stomach Adenocarcinoma	10.0	26.4	54.0	71.9
TCGA-SARC	Sarcoma	6.1	22.5	41.1	46.8
TCGA-COADREAD	Colorectal Adenocarcinoma	10.6	35.8	64.6	77.0
TCGA-CESC	Cervical Squamous Cell Carcinoma and Endocervical Adenocarcinoma	3.7	20.5	31.7	42.7
TCGA-HNSC	Head and Neck Squamous Cell Carcinoma	5.8	23.0	40.0	38.7
TCGA-BRCA	Breast Invasive Carcinoma	56.3	46.0	157.6	116.3

Supplementary Table 6. Comparison of accuracy (%) in forwarding to 1st layer, 2nd layer, and last layer to generate embeddings.

Cancer dataset	Cancer type	1st layer	2nd layer	last layer
TCGA-LGG	Brain Lower Grade Glioma	**99.06**	**99.06**	65.09
TCGA-UCEC	Uterine Corpus Endometrial Carcinoma	**84.31**	83.33	71.57
TCGA-STAD	Stomach Adenocarcinoma	**83.33**	80.77	71.79
TCGA-SARC	Sarcoma	69.39	**79.59**	**79.59**
TCGA-COADREAD	Colorectal Adenocarcinoma	61.54	64.84	**69.23**
TCGA-CESC	Cervical Squamous Cell Carcinoma and Endocervical Adenocarcinoma	97.30	**100.00**	86.49
TCGA-HNSC	Head and Neck Squamous Cell Carcinoma	**98.04**	96.08	92.16
TCGA-BRCA	Breast Invasive Carcinoma	**84.26**	79.70	68.02

Supplementary Figure 1. Initial GCN prediction accuracy across various threshold values for each omics (top left: CNA, top right: methylation, bottom left: mRNA, and bottom right: RPPA) from TCGA-LGG dataset. The x-axis shows the percentage of retained connections in the affinity matrix, and the y-axis displays the model accuracy.

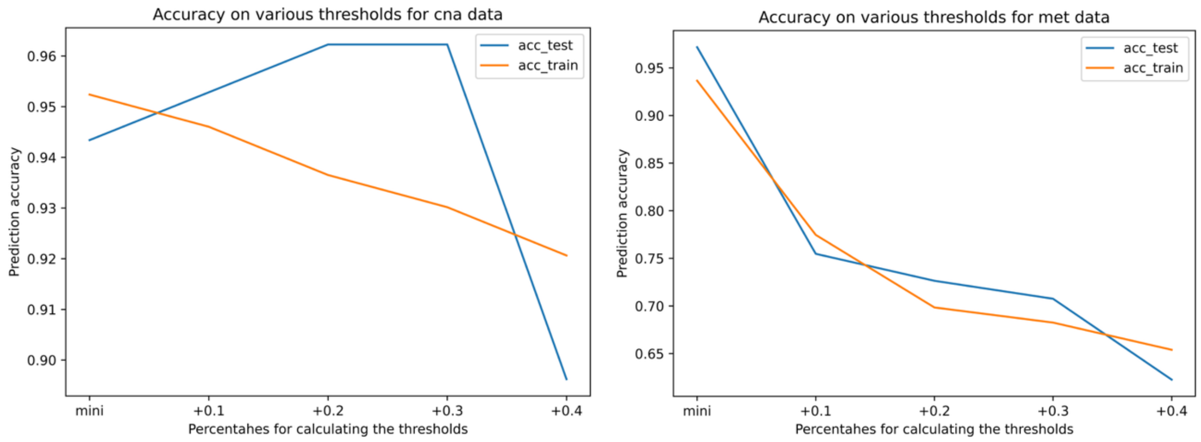

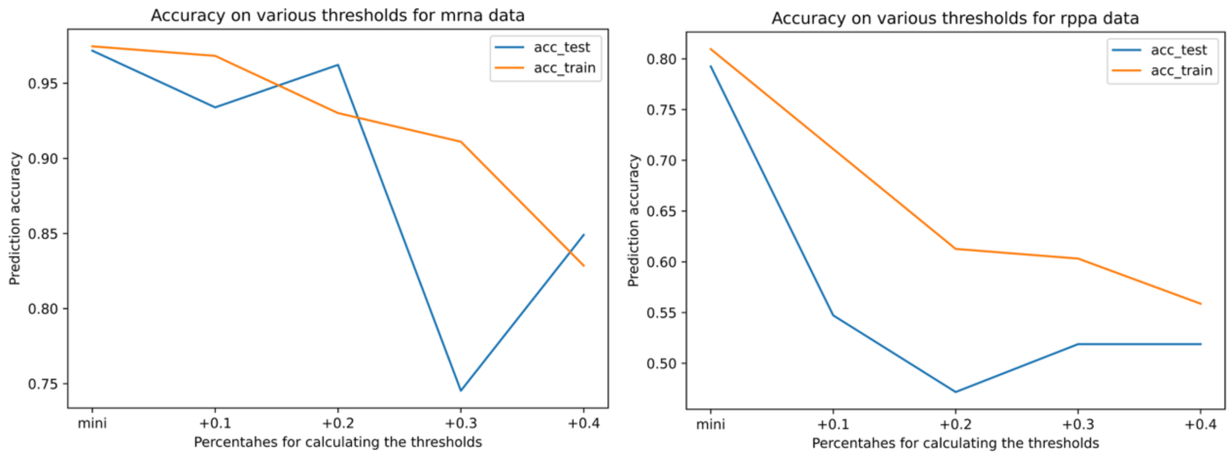

Supplementary Figure 2. Performance of the final GAT prediction model with a series of thresholds for TCGA-LGG dataset

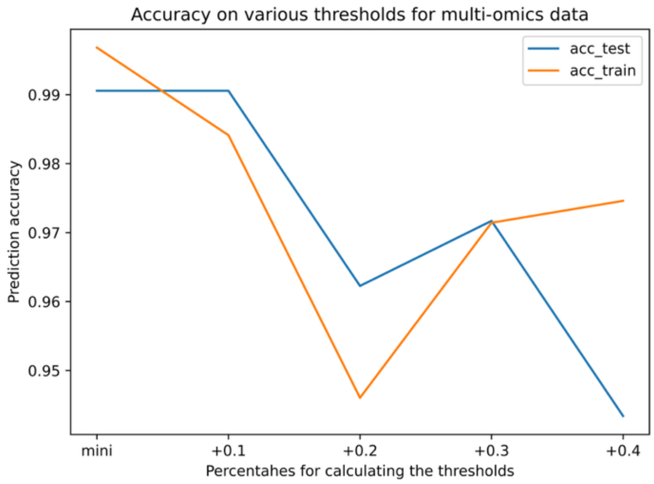

Supplementary Figure 3. Confusion matrices generated by MO-GCAN for each cancer type.

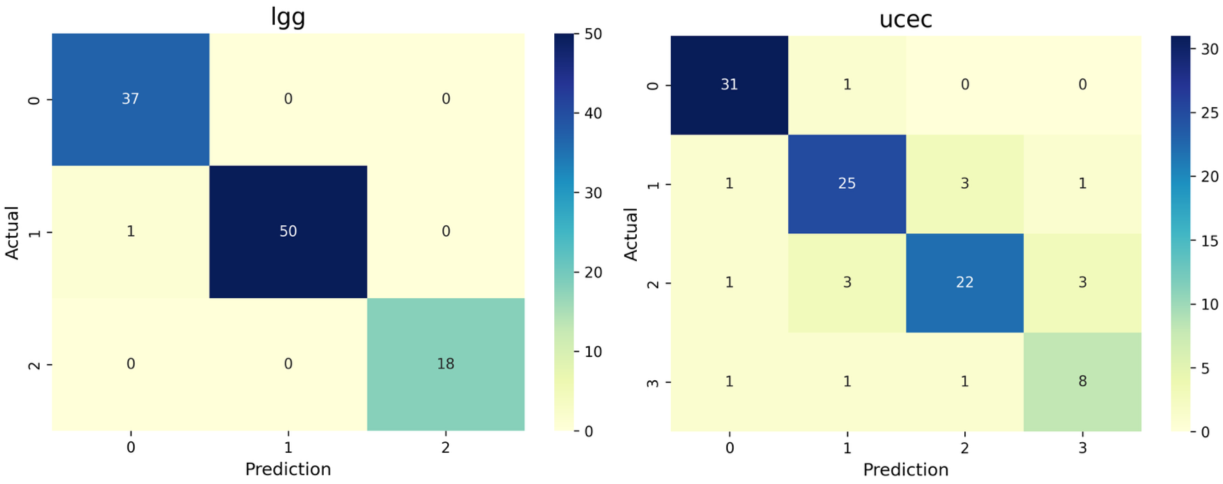

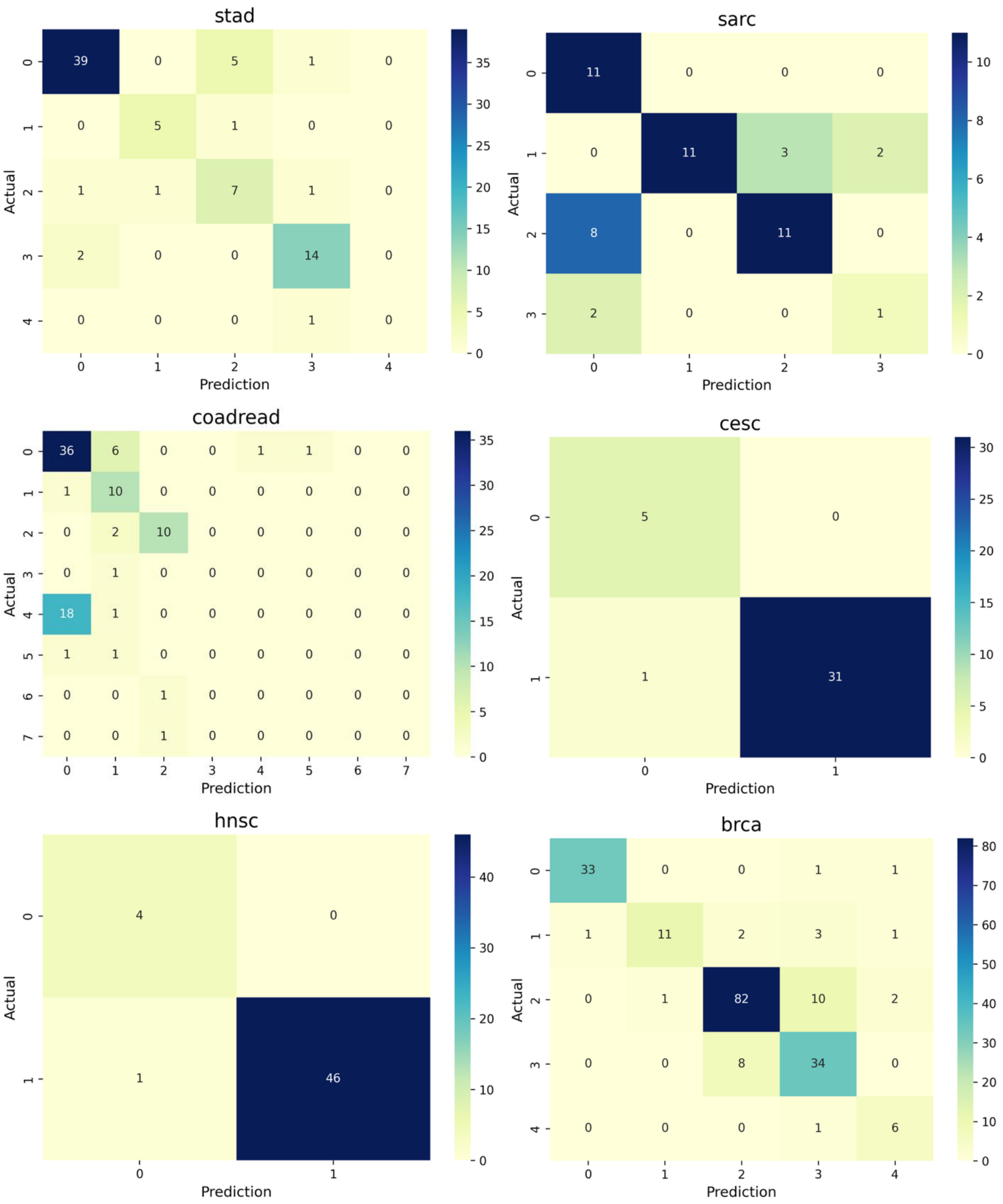
stad
Actual
Prediction
sarc
Actual
Prediction
coadread
Actual
Prediction
cesc
Actual
Prediction
hnsc
Actual
Prediction
brca
Actual
Prediction
